# Mentalising and conversation-following in autism

**DOI:** 10.1177/1362361320935690

**Published:** 2020-07-18

**Authors:** Hsuan-Chen Wu, Francesca Biondo, Ciara O’Mahony, Sarah White, Flora Thiebaut, Geraint Rees, Paul W Burgess

**Affiliations:** University College London, UK

**Keywords:** autism, conversation, heterogeneity, mentalising, social interaction

## Abstract

Some people with autism spectrum disorders have been observed to experience difficulties with making correct inferences in conversations in social situations. However, the nature and origin of their problem is rarely investigated. This study used manipulations of video stimuli to investigate two questions. The first question was whether it is the number of people involved in social situations, that is, the source of problems in following conversations, or whether it is the increased mentalising demands required to comprehend interactions between several people. The second question asked was whether the nature and pattern of the errors that autism spectrum disorder participants show are the same as typically developing people make when they make an error. In total, 43 typically developed adults and 30 adults diagnosed with autism spectrum disorder were studied. We found that it was the amount of mentalising required, rather than the number of people involved, which caused problems for people with autism spectrum disorder in following conversations. Furthermore, the autism spectrum disorder participants showed a more heterogeneous pattern of errors, showing less agreement among themselves than the typically developed group as to which test items were hardest. So, fully understanding the observed behaviour consequent upon weakness in mentalising ability in people with autism spectrum disorders requires consideration of factors other than mentalising.

People with autism spectrum disorders sometimes report difficulties with following observed conversations in social situations, especially those where several people are interacting with each other. But this has rarely been investigated directly. This study determines whether people with autism spectrum disorders do indeed have problems following observed conversations even when they perform well on IQ tests and investigates two possible reasons for any difficulty found: (1) some people may have a problem integrating stimuli from multiple speakers; (2) following a conversation between many people might make particularly high demands on mentalising abilities. We used a variety of video clips of people conversing together to investigate these two possibilities in 30 adults diagnosed with autism spectrum disorder and 43 age- and IQ-matched typical-developing adults. We found that it was the amount of mentalising required, rather than the number of people involved, which caused problems for people with autism spectrum disorder in following conversations. Furthermore, when the autism spectrum disorder participants made a mistake, the error they made was frequently not the same error that typically developed participants made, and the autism spectrum disorder population made a more varied set of errors than the typically developed participants. Together, these results suggest that people with autism spectrum disorders observe significant problems with following conversations between many people when they contain a lot of mentalising material, but where they do make a mistake, the conclusions they draw from the conversation they are observing may have a more complex cause than an impairment in mentalising alone.

## Introduction

This study arose out of a discussion that the authors had with Professor David Skuse (of University College London (UCL), London, UK) in 2013. Professor Skuse, who is a very experienced clinician and researcher in the field of autism (e.g. Skuse et al., 2004), was asked which everyday situations presented greatest difficulty for people with high-functioning forms of autism spectrum disorders (ASDs) – or at least, were the most commonly reported by them to him – but for which there was no standard objective clinical test. One of the situations that Prof. Skuse described was where a person with ASD symptoms is trying to follow a conversation between several people. His suggestion was that following a conversation between several people was often reported as harder by people with ASD than understanding a conversation between just two people. Note that the observation was not that the ASD person had more difficulty in *contributing* to the conversation, but in extracting the same meaning from it that those without ASD symptoms do. This difficulty – if it existed – was unlikely be due to the weakness in fundamental aspects of language, such as auditory–verbal comprehension, since the observation related only to people with ASD who scored well on, for example, IQ tests and those used to detect aphasia. This left two most obvious possible explanations for this phenomenon, if Prof. Skuse’s observation was true. The first would be that the problems were related to the increased mentalising demands made by several people interacting at once. The second plausible account is an ‘executive function’ explanation; that is, the person experiences increased distraction when there are multiple sources of information (i.e. several people talking) which requires rapid switching between them and integration of different information streams. So, the following study was designed to try to answer two simple questions: (1) Do people with ASD experience more difficulty understanding conversations that they observe when there are several people speaking to each other, than they do when only two people are talking? (2) If they do experience more difficulty, is this related to an increase in mentalising demands or is it related to the number of people speaking? Remarkably, perhaps, given the simplicity of these questions, and everyday life observations that relate to conversation-following (e.g. Thede & Coolidge, 2007), we could find no naturalistic study which directly addressed the observation as to whether high-functioning people with ASD find following conversations of groups of people more difficult than following discussions between two people. Contrasting with this relative lack of direct experimental evidence, there are, however, training programmes aimed at people with ASD that address conversational issues (e.g. Laugeson et al., 2012), and there are numerous discussions within the autism community about the issues relating to conversations. Many of these relate to difficulty in contributing to conversations, but some relate personal experiences with conversation-following along the executive function versus mentalising type distinction presented above. For instance, in 2016, on an online forum aimed at people with autism (https://www.autismforums.com/threads/conversations-with-people.17473/), one contributor wrote ‘I cannot hold a conversation if there’s a lot of background noise as I simply can’t hear above the din, and I find myself zoning out if there are a lot of conversations going on at once’. Another reports ‘I have a really difficult time making small talk or have conversations when the talk turns in a direction I hadn’t planned for. It’s like hitting a brick wall in my head’. One might speculate, perhaps, that the first description describes the issue relating to attentional or executive-type difficulties, and perhaps the second more of a mentalising one.

Despite a lack of direct naturalistic evidence, there is nevertheless a wealth of experimental studies that can speak to this matter. So, let us consider, in turn, the plausibility of the two main putative explanations for any ASD/TD difference in conversation-following, if observed, on the basis of this evidence.

First, considering the ‘mentalising hypothesis’, the awareness that other people have beliefs and desires different from our own is termed ‘theory of mind’ (ToM; Premack & Woodruff, 1978), and the term ‘mentalising’ has been used to refer more broadly to our ability to make inferences about our own and other people’s states of mind through processing interpersonal communications or subtle social cues, such as facial expression, voice tones, and body movements (Frith and Frith, 1999, 2006). A variety of different experimental paradigms have been used to investigate the underlying processes of mentalising. Given that narratives in movies often include entertaining elements showing interplays between characters’ mental states, the use of such materials has been an obvious place to start (Dziobek et al., 2006; Golan et al., 2007; Heavey et al., 2000). For example, ‘the Awkward Moments’ test (Heavey et al., 2000) used commercial videos depicting social scenes filled with complex visual and auditory information that highlighted the advantage of video stimuli, including subtle, transient social cues. However, there are some methodological considerations in the use of such material. For instance, commercials might not depict a particularly realistic or a rather exaggerated use of social cues compared with normal daily situations. Accordingly, Golan et al. (2007) developed the ‘Reading the Mind in Films’ (RMF) test, which consists of short scenes taken from feature films that relate to everyday life. Both ‘the Awkward Moments’ and the RMF tests were, however, similar in that the questions that asked about the video stimuli involved emotional adjectives (e.g. ‘embarrassed’, ‘shocked’ and ‘awkward’). This has the potential for introducing a confound in terms of measuring mentalising ability due to individual differences in interpretations of the meanings to those emotional adjectives, rather than measuring sensitivity to differences in interpreting states of mind per se. It is therefore important to be cautious in the design of both the nature of the stimuli, as well as the questions asked during the testing phase in experimental paradigms measuring mentalising, and we have been mindful of that here.

Since the landmark study of Baron-Cohen et al. (1985) reported that children with ASD showed impairments on ToM, several theories have emerged about the possible causes. These include a defective meta-representation (Baron-Cohen et al., 1985), deficits related to integration between the concept of ‘self’ and the social world (or the simulation theory; Goldman, 2006), problems with language comprehension affecting pragmatic inference (Loukusa & Moilanen, 2009), problems with emotional recognition that prevent complex perception of facial states (Castelli, 2005) and a potential indirect influence from executive dysfunction (see Hill, 2004; Robinson et al., 2009). On this account, one area where mentalising problems might show themselves is difficulties with following conversations, especially between groups of people, because there are more states of mind to track.

The second putative account we consider here is that the source of these problem lies with a difference in the basic attentional processes that allow us to attend to each particular speaker at any one time (e.g. Mesgarani & Chang, 2012). A prediction of this account would be that conversation-following by people with ASD symptoms would be poorer when many people are being observed rather than just two, independent of the content or topic of the conversation. Of course, it may be that it is more difficult for anyone, whether or not they show ASD symptoms, to follow many people conversing together. Hence, for this account to be supported, the ASD participants would need to show a disproportionate decrement in performance relative to IQ- and age-matched typically developed (TD) controls.

The third theoretical concern about studying abilities relating to following interpersonal conversation among ASD population is the definition of being ‘correct’, or more importantly, how to characterise the nature of conclusions people make about what they have heard are ‘not correct’. Therefore, in this study, we try to study the nature and pattern of errors rather than only the occurrence of them, which is rarely discussed in the literature. If a person is struggling to fully comprehend the dynamics of the conversation, it is possible that they will decide that they have no idea at all about the meaning of the interaction. However, this is only one possibility. It is also possible that they may come to a conclusion about what they have witnessed, despite the weak evidence that they have available to them. In terms of everyday consequences, this conclusion is likely to be quite an important determinant of the judgement of competence that an observer would make. If the error is similar in kind to many that the TD people might make, then the occurrence of the error might seem less noteworthy than if it is an error that rarely occurs in the TD population. Therefore, the form of the error is an important indicator of performance. It may also give some indication of the nature of the problem, for instance, that the responses are made impulsively, or too much attention being given to some aspect of the situation witnessed. One way of studying responses made under situations of uncertainty, which occur where a participant is asked to answer a question where they may not be sure of their answer, is to use signal detection methods. This approach was applied by Thiébaut et al. (2016) to the study of 43 TD adults and 35 adults with ASD on a test of social faux pas detection that used a cartoon format. Adults with ASD actually over-detected faux pas (i.e. thought a faux pas had been committed where the TDs did not), despite good comprehension abilities. Signal detection analysis indicated that the ASD participants had greater difficulty detecting whether a cartoon depicted a faux pas and showed a liberal response bias. Analysis of performance item-by-item revealed that the ASD group was not in agreement with a reference control group about which non-faux pas items were most difficult. Thiébaut et al. (2016) concluded that the participants with ASD had a primary problem with faux pas detection, but that there is another factor at work, possibly compensatory, that explained their choice of a liberal response criterion. In this way, to conclude that the ASD participants’ weak faux pas abilities alone explained their performance would have been to ignore an important determinant of the observed behavior, the most likely experimental prediction from the suggestion that the ASD participants might have poorer faux pas detection abilities would be increased frequency of errors of omission not of commission (i.e. they would say that fewer stimuli depicted faux pas, compared to TD controls, rather than more, which is what they actually did). In the same way, perhaps, describing complex misunderstandings from listening to conversations between people as resulting merely from an absence of some construct (such a mentalising) risks missing an important secondary determinant of behaviour.

Another recent study which demonstrates the importance of analysis of patterns of errors rather than only the number or frequency of them is given by Wu et al. (2018). They administered a gambling paradigm to a group of high-functioning adults who had diagnoses of ASDs and TD control participants who were matched for age and IQ. The ASD participants were no more or less likely to take a risk than the TDs. But they were more consistent in their choices from trial to trial, and the proportion of participants who always chose either the riskiest or most ‘safe’ option was significantly higher in the ASD group compared with the controls. This result showed the pitfalls of only averaging across participants as a way of characterising individuals’ behaviour: the average risk rates between the ASD and TD participants were not significantly different. But this did not mean that their behaviour was the same; the groups were achieving a similar rate overall, but in very different ways.

So for these reasons, we structure the current experiment in a way that permits examination of patterns of ‘errors’ to determine if the ASD participants, when they are incorrect, are incorrect in the same way as TDs.

Overall, we can formalise the hypotheses of this experiment in the following way:

*Hypothesis 1*: We assume it likely that everyone (i.e. both ASD and TD participants) will find following a conversation between multiple people harder than between only two, given that other factors, such as comprehensibility, linguistic difficulty, volume, and so on, are held broadly equivalent. This will be principally because the information load and attending requirements presented by several characters interacting will be greater.
*Hypothesis 2*: ASD participants will make more mistakes and take a longer time to respond when answering questions about the video clips than the age- and IQ-matched TD control participants. This reflects the suggestion that people with ASD have trouble extracting the meaning when watching discussions between people, or extract different meanings from them because these are inherently social situations, even if they do not require mentalising.
*Hypothesis 3*: The third hypothesis is that people with an ASD diagnosis will find questions about discussions that have occurred between several people harder to answer (i.e. will take longer to give answers and make more errors), relative to age- and IQ-matched TD controls than those that ask about two people’s verbal interactions.
*Hypothesis 4*: People with an ASD diagnosis will be especially poor when being asked about video clips that portray social situations that contain a lot of ‘mentalising’ content. This hypothesis follows from the suggestion that people with ASD have trouble processing social material with a high theory of mind and intentionality content.
*Hypothesis 5*: ASD participants will show a different *pattern* of errors from the TDs, making different responses (in type, number or proportion) when they make an error that TDs do when they make an error.

There are several ways in which such a hypothesis can be tested. The first is to consider whether the nature of the errors made to each test item is similar between the groups (Hypothesis 5(i)). Another way is to consider whether the relative proportions of error types across the entire pool of test items are similar (Hypothesis 5(ii)). A third way is to consider how frequently each individual test item is failed by each group; in other words, do ASD and TD participants show agreement as to which items are hard and which are easy? (Hypothesis 5(iii)).

To examine these five experimental hypotheses, we developed a video mentalising paradigm that (1) systematically manipulated the nature of the video stimuli we used and (2) implemented a standardised approach to measure mentalising competence in the testing phase. At the same time, we manipulated the number of characters involved in the video stimuli. Previous ToM studies have used different numbers of characters in depicted social scenes; for example, two characters were involved in the ‘Sally–Anne’ test (Baron-Cohen et al., 1985), multiple characters were involved in the ‘Awkward Moments Test’, and one to four characters were involved in the RMF test. By definition, social interaction between individuals involves at least two characters. However, it is possible that the number of characters involved above might introduce a systematic effect on mentalising demand. In other words, the amount of social information required to process increases as the number of the characters increase. Accordingly, we labelled the videos showing social interaction involving only two characters as ‘dyad’ videos, and the videos depicting social interaction involved more than two characters (three, four or five characters) as ‘multiple’ videos and crossed this factor experimentally with the degree of mentalising (high or low). As a result, video stimuli in the study each belonged to four categories: high mentalising + dyad (HD), high mentalising + multiple (HM), low mentalising + dyad (LD) and low mentalising + multiple (LM) videos. In defining which could be considered ‘high- mentalising’ video clips, we considered whether, to comprehend the conversation and answer the question about it, it was necessary to appreciate the characters’ feelings, thoughts and intentions. These could be inferred both through non-verbal behaviours and linguistic aspects of the conversation, including paralanguage. Thus, the high-mentalising videos featured material which invited or contained second-order false beliefs, pretence, deception and non-literal utterances and depicted everyday situations, such as a mother making poor excuses for not attending her daughter’s important event, an employer trying to be tactful when firing his employee, a female employee trying to flirt with a customer and a love triangle between two men and a woman. By contrast, the low-mentalising videos included scenarios, such as a policeman checking the licence of a driver, a show host asking the recipe for a traditional Maltese pie, two men discussing the function of a Victorian theatre and a personal assistant helping two visitors to arrange their evening schedules. In these low-mentalising clips, the conversation was largely an exchange of factual information, and it was not necessary to make any reference to the mental states of the characters to answer the multiple-choice questions (MCQs) that followed the clips. (Further information about test design and how the low- vs high-mentalising distinction was determined is given below.) These MCQs were designed to follow a standardised format using the same stem question for each test item throughout the test; only the multiple-choice options changed from clip to clip.

To investigate the potential source of any errors, we used a structured set of MCQ options across all test items. This was based on the principle of *appropriateness* as proposed by Castelli et al. (2000). Where participants chose the ‘correct’ MCQ option (equivalent to Castelli et al’s *appropriate* category), it indicated that the participant had made a correct inference about the intentions or emotional states of the characters and also understood the core topic of the social scenario. The second type of MCQ foil we employed is termed the ‘plausible’ option (equivalent to Castelli et al’s *partially correct answer*). This option represents choosing a response which relates to correct inferences about intentions or emotional states, but where there is confusion about the overall social situation. The ‘incorrect’ option (equivalent to *inappropriate answer*) meant that participants made wrong inferences about the intentions or emotional states. We added to these categories a series of foils that we term the ‘irrelevant’ option. The ‘irrelevant’ response options did not relate to the narrative of the scenario but included only keywords appearing in the conversation. Finally, we also recorded ‘no responses’ (equivalent to *no answer*), which was where participants were unable to provide any response within the time limit. These four response types (plausible, incorrect, irrelevant and no response) allowed us to analyse the characteristics of the responses which were NOT correct and to try to determine their root cause. Examples of the response options are given in Tables 1 and 3. As outlined above, this procedure allowed us to investigate whether people with ASD only fail to understand people’s states of mind, or whether they also extract different meanings from the material, or make different decisions about the material than do TD individuals. In other words, under the circumstances where ASD participants are unsure about an item, do they follow the same procedures and make the same choices as TD people? (see Burgess & Shallice, 1996, for a similar approach applied to frontal lobe lesioned neurological patients using the Hayling Sentence Completion test).

**Table 1.** An example of the four options, along with their statements, the response category they belong to, and explanations of their category for one typical test item. The scene depicted a mother visiting her daughter unexpectedly, as a pretext for making a feeble excuse for not attending her daughter’s forthcoming fashion show, which her daughter wanted her to see.

MCQ option	Statement	Category	Explanation
1	The daughter is disappointed to learn the purpose of her mother’s visit.	Correct	The daughter was able to successfully work out her mother’s true intention for her visit by hearing the hesitation during the conversation and gave her mother a judgmental look at the end.
2	The daughter is irritated because her mother is interfering with her work.	Incorrect	The daughter did show an irritated expression, but it was because of the feeble excuse, not the unexpected visit.
3	The daughter is sad that her mother cannot come to see the show.	Plausible	The daughter realised that her mother would not come to her important event, but the dramatic effect was mainly about the daughter working out the true reason behind her mother’s visit.
4	The daughter is irritated that she has to plan the wedding invitations.	Irrelevant	The mother did use a wedding as an excuse for her absence, but that is not the reason why the daughter reacted as she did.

MCQ: multiple-choice question.

## Method

### Participants

This study was approved by the UCL Research Ethics Committee (ID No.: 3825/001), and all individuals provided their informed consent to participate. In total, 43 TD participants (24 male) and 30 ASD participants (20 male) were recruited from the Institute of Cognitive Neuroscience participant database. All participants were aged between 18 and 70 years, native English speakers with no histories of hearing, visual or motor impairments. All ASD participants had clinical diagnoses, and all TD participants reported no psychiatric or neurological disorders, and none reported any ASD diagnoses among their first-degree relatives. In the ASD group, 9 were diagnosed with autism, 21 were diagnosed with Asperger’s syndrome by qualified clinicians in accordance with standard diagnostic criteria. The criteria included the Autism Diagnosis Observation Schedule (ADOS; C. Lord et al., 2000) for autism spectrum or autism, and/or the Autism Spectrum Quotients (AQ; Baron-Cohen et al., 2001). ADOS scores were available for 28 of the 30 ASD participants and 26 of them met the criteria for an ASD. The two participants whose ADOS scores fell below the cut-off score and the two ASD participants without ADOS score were not excluded as they provided reliable written clinical diagnosis, and their AQs were all above the recommended cut-off score of 32. All participants had full-scale Wechsler Intelligence Quotients (FSIQ) greater than 80 (WAIS-III-UK, Wechsler, 1998; WASI, Wechsler, 1999). The ASD and the TD groups were matched for age (*t*(71) = 1.041, p = 0.301), gender (*χ*^2^(1) = 0.869, p = 0.351), verbal IQ (*t*(71) = 0.598, p = 0.552) and performance IQ (*t*(71) = −0.416, p = 0.679) (see Table 2).

**Table 2.** Characteristics of participants.

	TD (*N* = 43)	ASD (*N* = 30)	*p* value^a^
Age (years), *M* (*SD*)	33.7 (9.3)	35.7 (10.5)	0.301
Male, *N* (%)	24 (56)	20 (67)	0.351
VIQ, *M* (*SD*)	114.42 (13.2)	116.07 (15.0)	0.552
PIQ, *M* (*SD*)	112.07 (12.9)	111.33 (14.3)	0.679
ADOS,^b^ *M* (*SD*)		8.2 (3.4)	
AQ,^c^ *M* (*SD*)		35.7 (9.3)	

TD: typically developed; ASD: autism spectrum disorders; VIQ: Wechsler Intelligence Scale Verbal Intelligence Quotient; PIQ: Wechsler Intelligence Scale Performance Intelligence Quotient; ADOS: Autism Diagnosis Observation Schedule; AQ: Autism Spectrum Quotients.

a Independent *t*-test or Fisher’s exact test, two-tailed, for significant differences between groups.

b Autism Diagnostic Observation Schedule; *n* = 28.

c Autism Spectrum Quotients; *n* = 30.

### Experimental design

This study employed a 2 (high or low mentalising) × 2 (dyad or multiple characters) factorial design. The video mentalising test was displayed using DMDX experimental software (Forster & Forster, 2003) and consisted of 14 coloured short video clips taken from BBC television programmes, including two that were used for practice. The video clips included both visual (facial expression, body language and physical interactions) and auditory inputs (verbal content and intonation changes) depicting various kinds of daily social interactions. The video clips were categorised into HD, HM, LD and LM categories, according to the level of mentalising required (high vs low mentalising) and the numbers of characters involved (dyad vs multiple). For the ‘characters’ factor, dyad videos included exactly two characters in the scene, and multiple videos depicted scenes included three to five (*M* = 3.75 ± 0.7) characters.

### Mentalising test design and development

As outlined above, we manipulated the nature of the video stimuli along two-factor dimensions: the level of ‘mentalising’ required to process the stimuli and the number of ‘characters’ involved in the scene. A sample of 64 test items (film clips) was initially created. All the film clips featured conversations in a range of settings (e.g. home, work, leisure) and featured a conversation which did not necessitate prior knowledge of the characters or plot for correct comprehension. A priori ratings of the degree of mentalising required to follow the action depicted in the film clips and answer the questions (which differed in terms of the degree to which they referred to the states of mind of the characters portrayed) were made by three researchers, and the 64 items were divided into two samples of 32, with one sample representing the clips thought to make the highest mentalising demands and the other the lowest. The definition for ‘high mentalising’ was based on the ToM account of autism (Baron-Cohen et al., 1985) and followed two published descriptions of high- versus low-mentalising demands. The first was the principle used in the Strange Stories task (Happé, 1994), where the rater considered whether, to comprehend the depicted conversation, it was necessary to appreciate the characters’ feelings, thoughts and intentions which can be inferred both through the non-verbal behaviour and the linguistic aspects of the conversation, including paralanguage. High-mentalising clips featured second-order false beliefs, pretence, deception and non-literal utterances. By contrast, for the ‘low-mentalising’ items, the conversation was largely an exchange of factual information. Comprehending the dialogue relevant to the low-mentalising questions did not require any reference to the mental states of the characters, only requiring the ability to reason about physical properties and cause and effect; for example, ‘what do you think was the doctor’s order?’. The second rating principle was to consider how closely the questions asked about the low- and high-mentalising clips mapped onto the degree of intentionality as proposed by Castelli et al. (2000), as judged by the three raters (see Table 3 for examples). Castelli et al award a score between 0 and 5 for degrees of intentionality, with non-deliberate action (e.g. a person is moving his/her arm with no obvious purpose to it) assigned 0; deliberate action with no other person (e.g. a person ice-skating) assigned 1; deliberate action with another person (e.g. blue and red are fighting) awarded 2; deliberate action in response to other’s action (e.g. two people are arguing) given 4; deliberate action with the goal of affecting another person’s mental state given 5 (e.g. a child pretending not to be doing anything). In each case, those questions that required higher levels of appreciation of mental states (score 4–5 in Castelli et al., 2000) to answer occurred within the ‘high-mentalising’ trial videos, and those that required lower levels of appreciation of mental states (score 0–3 in Castelli et al., 2000) to answer occurred within the ‘low-mentalising’ video trials. In practice, this meant that the experimental questions for high-mentalising items tapped strongly into the mind-reading aspects of the conversation; for example, ‘what do you think was the woman’s intention?’ Examples are shown in Table 3.

**Table 3.** Two examples of the video mentalising test, including two different conditions, such as the experimental procedure and the response types of the questions, were listed.

Condition	Procedure		Response type
	Cue	3, 2, 1 . . .	
	Video	A scene depicting a mother making poor excuses for not attending her daughter’s fashion show.	
	Question	Which of these statements best describes the situation you have just seen?	
		1. The daughter is disappointed to learn the purpose of her mother’s visit.	Correct
		2. The daughter is irritated because her mother is interfering with her work.	Incorrect
HD condition		3. The daughter is sad that her mother cannot come to see the show.	Plausible
		4. The daughter is irritated that she has to plan the wedding invitations.	Irrelevant
	Question	Which of these statements best describes the situation you have just seen?	
		1. The mother really wants to see her daughter’s fashion show.	Incorrect
		2. The daughter thought her mother had come to see her show.	Correct
		3. The daughter secretly wants to offer her help on the seating plans.	Irrelevant
		4. The daughter has been expecting her invitation to the wedding for a long time.	Plausible
	Cue	3, 2, 1 . . .	
	Video	A scene depicting a new employee giving a brief summary of his CV to three colleagues before lunch.	
	Question	Which of these statements best describes the situation you have just seen?	
		1. The men will soon be working on a project together.	Plausible
LM condition		2. The men will soon be travelling together on business.	Irrelevant
		3. The men are going to have lunch together after the meeting.	Correct
		4. The men had been introduced before this meeting.	Incorrect
	Question	Which of these statements best describes the situation you have just seen?	
		1. The young man who is introduced used to work for Mercedes-Benz.	Correct
		2. The young man who is introduced is currently looking for a job.	Irrelevant
		3. The young man who is introduced drives a Mercedes-Benz car.	Plausible
		4. The young man who is introduced used to work for Draper.	Incorrect

HD: high-mentalising dyad; CV: curriculum vitae; LM: low-mentalising multiple-person conversation.

These test items were piloted on a sample of TD adults not otherwise used in this study (UCL students and their friends). Each test item from each high- and low-mentalising sample of film clips was then analysed to determine how well it predicted the overall performance on the other items from that sample. For each sample (high and low mentalising), the items that best predicted the performance on the other items were retained, with the constraint that there needed to be a balanced number of high- and low-mentalising items, and those depicting conversations between either two or more than two people. This test development procedure reduced the initial test item pool from 64 to 12 (six high- and six low-mentalising items) video clips, plus two practice items.

Two MCQs were asked for each video clip. So the maximum score on this test was 24 (12 clips × 2 questions). The question frame for every MCQ question was the same: ‘which of these statements best describes the situation you have just seen?’ This was followed by four possible options in different categories (correct, plausible, incorrect and irrelevant; see Table 3 for examples from low- and high-mentalising items). All the options in the MCQs were matched for sentence length and readability. In the video mentalising test, each condition (HD, HM, LD and LM) contained three video clips, along with six MCQs. The order of the video clips was counterbalanced in a pseudorandom way and was identical across all the participants. The position of the correct answer within each MCQ item was also counterbalanced. A threshold for maximum reaction time (RT) of 27 s was determined as two standard deviations above the mean from a pilot study involving a separate sample of eight TD individuals.

### Procedure

At the beginning of the test, participants were asked to wear headphones and we adjusted the volume to a level with which the participant felt comfortable. The participants were then given instructions that explained the response keys and were given two practice videos with accompanying sets of MCQs. Participants were asked to choose the MCQ option that best described the scene depicted in the video by pressing the corresponding 1, 2, 3, 4 keys. There was a 3-s countdown, with a fixation cross, presented before the presentation of each video clip. The first MCQ appeared on the screen for 27 s after the presentation of each video (maximum exposure time determined by normative data collected during the test development stage). If the maximum permitted RT (27 s) was reached, a sign saying, ‘Time is up!’ was displayed for 300 ms on the screen and the next MCQ appeared on the screen. Under such circumstances, the response type would be registered as an ‘error’ and the RT would be registered as 27 s. The mentalising ability we try to examine here, as presented in video form, depicts interpersonal communications requiring an almost immediate response. Therefore, any delayed responses after a prolonged pause of action (i.e. 27 s as the pilot study suggested) would be viewed as atypical according to social convention. The next question was presented 300 ms after the response for the preceding MCQ response was registered. The video mentalising test took each participant approximately 20 min to administer. The responses and the RTs to each MCQ were recorded.

## Results

To test the first four hypotheses, measures of accuracy and RT were entered into three-way repeated measures ANOVA with mentalising (high vs low), characters (dyad vs multiple) as within-subject factors and group (TD vs ASD) as a between-subject factor (see Table 4 for summary). We will first consider accuracy, and then the response times.

**Table 4.** The mean (*M*) and standard deviation (*SD*) of accuracy and RT (seconds) in each experimental condition between groups in the video mentalising test.

Variable	Condition	TD			ASD			T-value	Cohen’s d
		*M*	*SD*	95% CI	*M*	*SD*	95% CI		
Accuracy									
	Overall	0.74	0.11	0.70–0.77	0.66	0.13	0.61–0.70	2.88 **	0.68
	HD videos	0.76	0.13	0.72–0.80	0.67	0.19	0.60–0.74	2.33 *	0.55
	HM videos	0.74	0.18	0.69–0.80	0.58	0.24	0.49–0.67	3.12 **	0.88
	LD videos	0.86	0.14	0.82–0.90	0.78	0.19	0.71–0.85	2.01 *	0.48
	LM videos	0.59	0.21	0.52–0.65	0.58	0.20	0.51–0.66	0.07	0.02
RT (s)									
	Overall	11.58	2.38	10.84–12.31	12.48	3.31	11.25–13.72	1.36	0.32
	HD videos	10.70	2.79	9.94–11.56	11.92	3.56	10.59–13.25	1.64	0.39
	HM videos	13.20	2.72	12.37–14.04	15.05	4.15	13.50–16.60	2.14 *	0.63
	LD videos	10.71	2.74	9.87–11.55	11.25	3.65	9.88–12.61	0.72	0.17
	LM videos	11.69	2.49	10.93–12.46	11.71	3.29	10.48–12.94	0.03	0.01

TD: typically developed; ASD: autism spectrum disorders; CI: confidence interval; HD: high-mentalising dyad; HM: high-mentalising multiple; LD: low-mentalising dyad; LM: low-mentalising multiple.

* p < 0.05, **p < 0.01.

### Accuracy

A three-way repeated measures ANOVA identified a significant main effect of the number of characters in the videos depicted (*F*(1,71) = 54.052, p < 0.001, *η*^2^ = 0.432, 95% CI [0.105, 0.184]) across all participants. This indicates that participants in both groups (TD and ASD) made significantly more correct responses to videos that involved only two characters conversing with each other than videos involving more than two. Thus Hypothesis 1 was supported.

Hypothesis 2 was that the ASD participants will make more errors than the TD participants when answering questions about the video clips, considered as a whole, compared to the age- and IQ-matched TD control participants. This hypothesis was also supported; there was a significant main effect of group (*F*(1,71) = 8.298, p = 0.005, *η*^2^ = 0.105, 95% CI [−0.139, −0.025]) indicating that the ASD participants made significantly fewer correct responses overall compared to the TD individuals. Although the mentalising × characters × group interaction just failed to reach p > 0.5 (*F*(1,71) = 3.781, p = 0.056, *η*^2^ = 0.056), follow-up analyses conducted for this marginal effect for exploration purposes revealed that compared with the TD group, the ASD group showed significantly lower accuracy in HD, LD and HM videos (all ps < 0.05), but the difference was not significant in LM videos (*t*(71) =−0.072, p = 0.943).

Hypothesis 3 is that people with an ASD diagnosis will find questions about discussions that have occurred between several (vs. only two) people harder to answer relative to age- and IQ-matched TD controls. This hypothesis was not supported by the accuracy data since repeated measures ANOVA found no significant character × group interaction (*F*(1,71) < 0.001, p = 0.997, *η*^2^ < 0.001). On average, the ASD participants made 5.00 errors (*SD* = 1.88) to the ‘multiple characters’ video clips, and 3.27 errors (*SD* = 1.66) to the dyad (i.e. two people conversing). By comparison, the TD group made mean 4.02 errors (*SD* = 2.13) to the ‘multiple characters’ video clips, and 2.28 errors (*SD* = 1.22) to the dyad (i.e. two people conversing).

Hypothesis 4 was that people with an ASD diagnosis will be especially poor when being asked about video clips that portray social situations that contain a lot of ‘mentalising’ content. Interestingly, no significant main effect of mentalising was found (*F*(1,71) = 0.528, p = 0.47, *η*^2^ = 0.007, 95% CI [−0.051, 0.024]). In other words, across *all* participants, high-mentalising items were not found more difficult to answer accurately than low-mentalising ones. But the critical test of Hypothesis 4 relates to the group differences. Here, a significant mentalising × group interaction was identified (*F*(1,71) = 5.000, p = 0.028, *η*^2^ = 0.066). Post hoc analysis confirmed that the ASD group made significantly fewer correct responses than the TD group only in response to the high-mentalising videos (*t*(71) = −3.785, p < 0.001, Cohen’s d = 0.90); the difference was not significant for the low-mentalising videos (*t*(71) = −1.137, p = 0.259, Cohen’s d = 0.27). Thus, Hypothesis 4 is supported.

### RTs

We will now consider the equivalent analyses in terms of the experimental Hypotheses 1–4 for RTs (i.e. the length of time between presentation of the questions and when the participant made a response). Before testing our hypotheses, the distributions of RTs are first examined. Analysis of the skewness in the four categories between groups revealed that only the RTs to HD video in the ASD group is highly skewed (skewness = 1.091, kurtosis = 1.962), and response latency to all the other categories, as well as the TD group, are all symmetrically distributed (<−0.5 <skewness < 0.5). The first hypothesis is that everyone (i.e. both ASD and TD participants) will find following a conversation between multiple people harder than between only two. Repeated measures ANOVA found a significant main effect of characters (*F*(1,71) = 62.020, p < 0.001, *η*^2^ = 0.466, 95% CI [−2217.253, −1321.322]) across all participants. These results indicate that across all participants, there was a tendency to take a significantly longer time to respond when the videos had involved multiple characters than where the videos had shown only two people conversing. Thus, Hypothesis 1 was supported by the results.

Hypothesis 2 was that the ASD participants will be slower to respond than the TD participants when answering questions about the video clips, considered as a whole, compared to the age- and IQ-matched TD control participants. This hypothesis was *not* supported: Repeated measures ANOVA did not find a significant main effect of group (*F*(1,71) = 1.853, p = 0.178, *η*^2^ = 0.025, 95% CI [ −420.821, 2231.518]; see Table 4). However, a caveat needs to be applied here; in our video paradigm, participants were time-limited (to 27 s) to give a response, based on piloting of TD people’s performances on the task. Accordingly, in the calculation of these statistics, trials where people failed to respond were coded as 27 s. Thus, if we had not put a time limit on responding, it is possible that we may have detected some out of (TD) range (i.e. atypical) values in the ASD group. However, after removing all the trials coded as 27 s because of a failure to respond, a repeated measures ANOVA still showed no significant main effect of group (p = 0.301, *η*^2^ = 0.015). This indicates that the ASD participants were not responding more slowly for trials other than ‘no response’ ones.

Hypothesis 3 was that people with an ASD diagnosis will take longer to answer questions about discussions that have occurred between several (vs only two) people relative to age- and IQ-matched TD controls. However, no significant characters × group (*F*(1,71) = 0.015, p = 0.904, *η*^2^ < 0.001) or mentalising × characters × group interactions (*F*(1,71) = 2.870, p = 0.095, *η*^2^ = 0.039) were found. The ASD participants were not significantly slower in responding to the questions about clips that depicted group situations than to clips showing two people (dyads), or in different experimental conditions.

Hypothesis 4 was that people with an ASD diagnosis will be especially slow to respond when asked about video clips that portray social situations that contain a lot of ‘mentalising’ content. And indeed, repeated measures ANOVA found a significant main effect of mentalising (*F*(1,71) = 57.458, p < 0.001, *η*^2^ = 0.447, 95% CI [1015.685, 1740.771]) and a significant mentalising × group interaction (*F*(1,71) = 11.982, p = 0.001, *η*^2^ = 0.144), thus supporting the hypothesis. Post hoc analysis confirmed that ASD participants took significantly longer than the TDs to respond to the high-mentalising videos (*t*(71) = 2.182, p = 0.032, Cohen’s d = 0.52), but that the group difference was not significant for low-mentalising videos (*t*(71) = 0.409, p = 0.684, Cohen’s d = 0.10).

### Analysis of error types

Hypothesis 5 involves the analysis of form, frequency and relative proportions of error types. The foregoing analysis makes it clear that the ASD group made more errors than the TD group. However, there are two main ways in which they might be different on this test. The first is that they show a similar pattern of errors but make more of them (i.e. a ‘similar but worse’ pattern). On this account, the ASD participants would tend to make errors most on the items that the TDs also found hardest, and when they did so, they would choose the same MCQ response options as the TDs. The second possibility is the ‘different and worse’ pattern. On this account, the ASD participants would make more errors than the TDs, but the MCQ response options they would choose when they made an error would be different (Hypothesis 5(i)).

**Figure 1.**
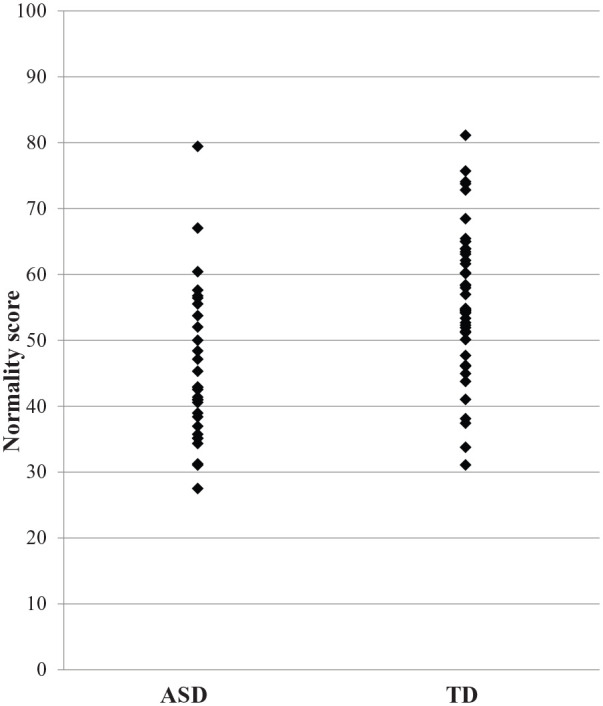
Scatter plot of normality score between groups.

To investigate which of these accounts best represented the error patterns, we examined whether the ASD participants showed a distinct response pattern that differs from the TD group using a weighted ‘normality’ score. First, for each test item, the proportions of each category of response types in the TD group were calculated, which indicated how unusual it would be for a TD individual to make each of the error types when shown each particular video clip (e.g. plausible, incorrect, irrelevant and no response; see Table 1). The proportions were divided by the total number of erroneous responses to make them independent from the number of correct responses. For example, if the TDs on test item 1, when making erroneous responses, made 60% errors in terms of ‘plausible’ options, 25% of the errors were ‘incorrect’ options, 10% errors belonged to the ‘irrelevant’ category and there were 5% of ‘no responses’; a weighted ‘normality’ score would assign 60 to the plausible option, 25 to the incorrect option, 10 to the irrelevant option and 5 to a no response in test item 1. For both TD and ASD participants, the summed ‘normality’ score would then be divided by their total number of erroneous responses to make the final ‘normality’ score independent of their ability to choose the correct option. The higher the ‘normality’ score a participant could get would indicate the more normal a response pattern a participant has. Independent *t-*test revealed that the ‘normality’ score in the ASD group was significantly lower than the TD group (ASD: *M* = 45.56, *SD* = 11.61; TD: *M* = 56.44, *SD* = 11.18; *t*(71) = −4.025, p < 0.001, Cohen’s d = 0.96; see Figure 1).

**Figure 2.**
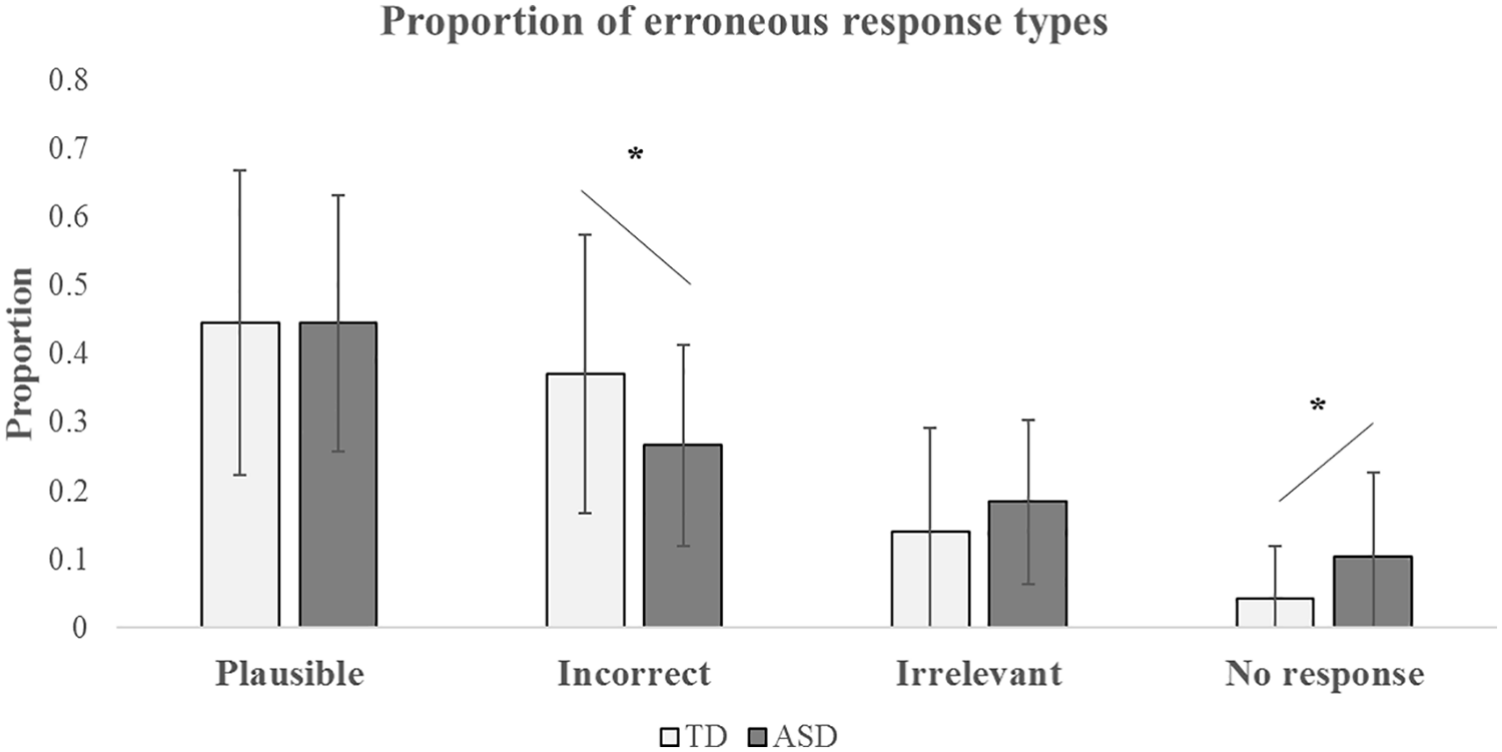
Proportion of the four categories of erroneous responses (plausible, incorrect, irrelevant and no response) between groups.

To identify further the nature of this atypical response pattern of the ASD group, we analysed which categories of erroneous responses showed significant differences between ASD and TD by calculating the proportions of the four categories (plausible, incorrect, irrelevant and no response) divided by the total number of erroneous responses (see Table 5 and Figure 2). Hypothesis 5(ii) is that this proportion of different error types will be significantly different between the TD and ASD groups. Independent *t*-test revealed significant group differences in ‘incorrect’ and ‘no response’ categories, where the ASD group has a significantly smaller proportion of ‘incorrect’ responses (*t*(71) = −2.38, p = 0.02, Cohen’s d = 0.56) and a significantly larger proportion of ‘no responses’ (*t*(71) = 2.588, p = 0.012, Cohen’s d = 0.61), compared with the TD group. The group difference in the ‘no response’ category remained significant (*F*(1,70) = 4.721, p = 0.033, *η*^2^ = 0.063) after co-varying for general RTs in case of a potential ceiling effect. But one of the most striking findings is a group × error type proportional difference between ‘incorrect’ and ‘irrelevant’ response choices. This is shown in Table 5. The TD participants made almost three times the absolute number of *incorrect* MCQ choices compared to *irrelevant* ones. But the ASD participants made only 25% more (*χ*^2^(1) = 8.392, p = 0.004).

**Table 5.** Percentage of errors of each type by group and experimental condition.

Erroneous response	TD					ASD				
	HD (%)	HM (%)	LD (%)	LM (%)	Sum (%)	HD (%)	HM (%)	LD (%)	LM (%)	Sum (%)
Plausible	15.8	9.0	4.5	15.0	44.3	14.2	11.3	4.1	14.2	43.8
Incorrect	2.6	9.8	6.0	19.2	37.6	2.0	7.3	6.1	10.5	25.9
Irrelevant	4.5	3.8	1.1	3.4	12.8	5.3	6.9	4.0	3.2	19.4
No response	0.4	1.9	1.1	1.9	5.3	2.0	4.9	1.6	2.4	10.9
Total					100.0					100.0

TD: typically developed; ASD: autism spectrum disorders; HD: high-mentalising dyad; HM: high-mentalising multiple; LD: low-mentalising dyad; LM: low-mentalising multiple.

Sum (%) refers to addition of each erroneous response across experimental conditions.

**Figure 3.**
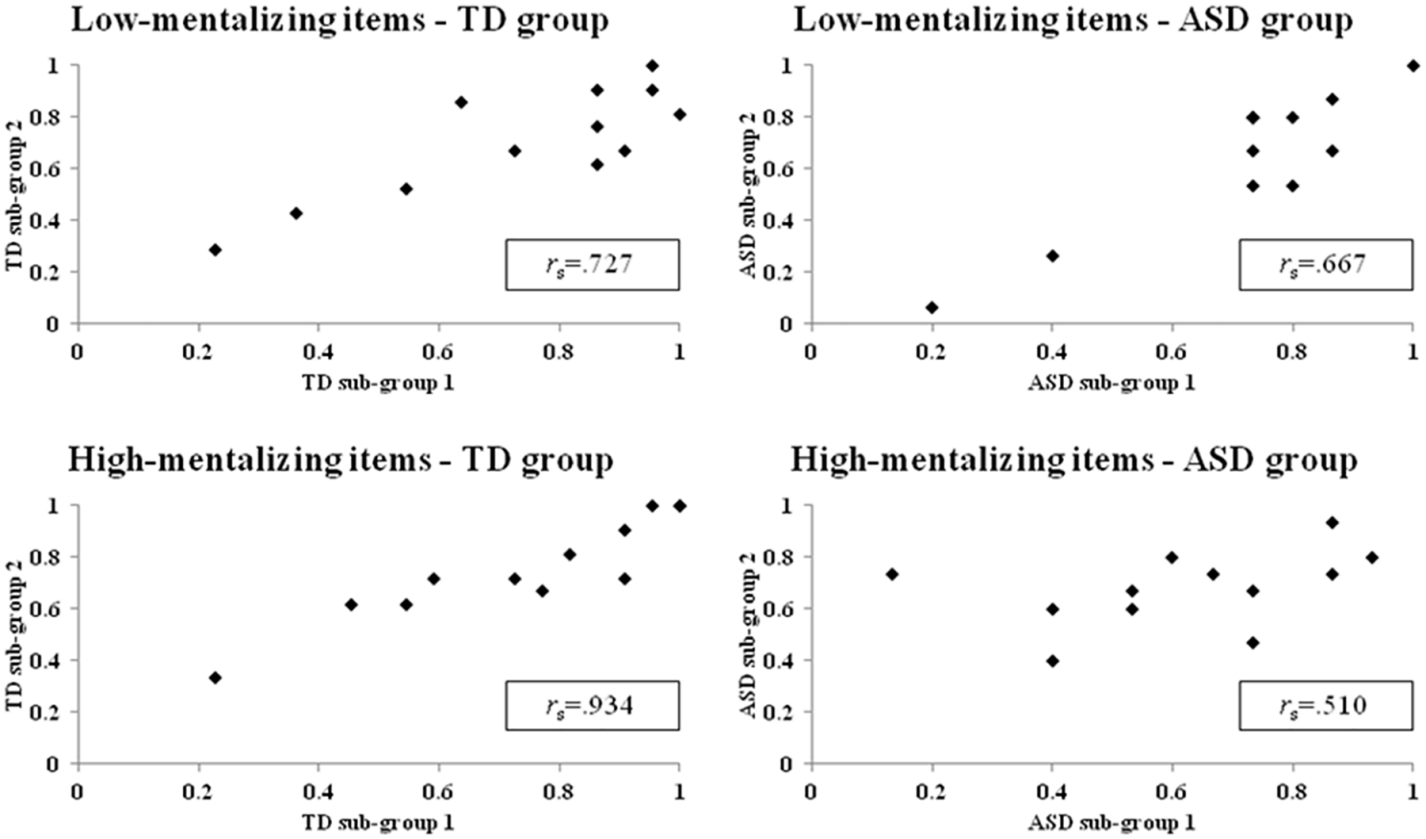
Spearman’s rank correlations of item difficulty in high- and low-mentalising items between the TD and the ASD subgroups.

Another approach to addressing the unusual ASD error pattern is to ask whether the ASD participants are different from the TD group, but similar to each other in terms of which items they find hard. Hypothesis 5(iii) is that this ‘item difficulty’ will not be in agreement across the TD and ASD groups. We investigated this by comparing *between-*groups with the degree of agreement of item difficulty of *within-*groups. To achieve this, we divided randomly the TD group into two subgroups and measured the agreement between the two groups about individual item difficulty by calculating the Spearman’s rank-order correlation coefficient among high- and low-mentalising items separately. The result revealed significant correlations between the two TD subgroups for both low-mentalising items (*r*_s_ = 0.727, p = 0.007) and high-mentalising items (*r*_s_ = 0.934, p < 0.001). In other words, there is a high degree of agreement among the TD participants as to which item is difficult (i.e. less likely to be responded to correctly). However, in the ASD group, Spearman’s rank-order correlation analysis showed a significant correlation for low-mentalising items (*r*_s_ = 0.667, p = 0.018), but not for the high-mentalising items (*r*_s_ = 0.510, p = 0.09; see Figure 3 for demonstration). The correlation coefficients between groups were converted into z-scores using Fisher’s r-to-z transformation to compute the statistical significance of the differences between two independent correlations. For high-mentalising items, the strength of the correlations between groups was significantly different (*z* = 2.389, p = 0.016, two-tailed), but the difference was not significant for low-mentalising items (*z* = 0.248, p = 0.803, two-tailed). This distinct pattern between high- and low-mentalising items remained the same after another random split of subgroups (high-mentalising items: *z* = 2.02, p = 0.04, two-tailed; low-mentalising items: *z* = 0.48, p = 0.631, two-tailed). Thus, there was more variability in the ASD group (vs the TDs) as to which of the high-mentalising test items they found difficult. Hence, Hypothesis 5(iii) was supported.

Perhaps noteworthy in interpreting these differences in patterns of errors are the RTs for the different types of response between the groups. For instance, if one group was merely guessing, or was being impulsive, or giving undue consideration before choosing a default response, one might expect to see RT differences associated with the different types of error. These data are shown in Table 6. For the TDs, the RTs for each of the error responses (other than ‘no response’) were very similar, with RTs to ‘plausible’ errors only slightly faster than to ‘incorrect’ or ‘irrelevant’ ones, which were almost identical. For the ASD group participants, RTs for the ‘plausible’ (*t*(69) = 0.406, p = 0.686, Cohen’s d = 0.10) and ‘incorrect’ (*t*(66) = 0.274, p = 0.785, Cohen’s d = 0.06) errors were very comparable to those of the TD group. However, RTs to the ‘irrelevant’ errors were somewhat longer, but this difference was not significant (*t*(43) = 1.837, p = 0.073, Cohen’s d = 0.56). These results therefore likely exclude a set of possible explanations for the error differences between the ASD and TD groups (e.g. that the TD participants made almost three times the absolute number of ‘incorrect’ MCQ choices compared to the ‘irrelevant’ ones, whereas the ASD group made only 25% more).

**Table 6.** RTs (in seconds) of errors of each type by group and experimental condition.

Erroneous response		TD					ASD				
		HD	HM	LD	LM	Overall	HD	HM	LD	LM	Overall
Plausible	*M*	10.6	13.6	13.4	12.9	12.3	11.1	13.7	14.9	13.0	12.8
	*SD*	3.2	3.6	5.2	4.7	4.2	3.3	4.6	5.8	4.2	4.3
Incorrect	*M*	10.7	14.8	13.2	12.6	13.1	12.8	13.0	11.6	12.4	12.4
	*SD*	5.9	2.4	5.0	3.7	4.1	6.9	5.3	5.0	3.4	4.6
Irrelevant	*M*	12.1	15.9	13.3	10.4	13.1	14.7	16.3	14.8	13.4	15.0
	*SD*	4.9	4.5	6.2	3.0	4.8	5.5	6.3	7.3	4.9	6.0
No response^a^	*M*	27.0	27.0	27.0	27.0	27.0	27.0	27.0	27.0	27.0	27.0
	*SD*	0.0	0.0	0.0	0.0	0.0	0.0	0.0	0.0	0.0	0.0
No. of no responses (*M, SD*)						0.33^b^ (0.57)					0.90^c^ (1.24)

TD: typically developed; ASD: autism spectrum disorders; HD: high-mentalising dyad; HM: high-mentalising multiple; LD: low-mentalising dyad; LM: low-mentalising multiple.

Overall refers to the RTs for each erroneous response across conditions

a The maximum time allowed for any one trial was 27 s, so ‘no responses’ were coded as 27 s (but the pattern of results reported in the text does not change substantively if these trials are removed from the data).

b The number of people in the TD group who produced 0, 1, 2, 3, 4 or 5 ‘no responses’ were 31, 10, 2, 0, 0, 0, respectively.

c The number of people in the ASD group who produced 0, 1, 2, 3, 4 or 5 ‘no responses’ were 14, 11, 2, 1, 1, 1, respectively.

## Discussion

The results of this investigation are summarised in Table 7 and suggest two broad conclusions in particular. The first is that where high-functioning people with a diagnosis of autism report difficulties with following conversations between people, the root cause of this is more likely to be that multiple people means multiple minds to read, rather than the root of the problem being, for example, the increased attentional or language comprehension demands of multi-person interactions, or problems with basic sensory processing. Both TD and ASD participants generally found it harder to follow conversations between several people (compared to just two), but the ASD participants were not significantly more susceptible to this than the TD participants. Prima facie this might be surprising given recent growing evidence of differences in basic sensory processing in some people with ASD, any of which might affect the performance when listening to complex conversations (Lawson et al., 2015) in naturalistic situations. In this respect, it is possible that the professionally edited and presented video clips that we used, where the direction of attention is somewhat determined by the director and editor, do not fully tap all the mental processes required for conversation-following in ‘real life’. However, these results nevertheless might suggest the relative importance of mentalising abilities versus other ones in relation to conversation-following.

**Table 7.** Summary of hypotheses and results. ‘Yes’ indicates that the hypothesis was supported by the data collected. ‘No’ indicates that the hypothesis was not supported.

Hypothesis	Summary of hypothesis	Accuracy	Speed
1	All participants find multi-person conversations more difficult.	Yes	Yes
2	ASD will be worse than TD on all test items.	Yes	No^a^
3	ASD will find multi-person interactions more difficult than dyads.	No	No
4	ASD will find high-mentalising clips harder than TD.	Yes	Yes
5	(i) ASD and TD make different errors to individual test items.	Yes	
	(ii) The proportion of different error types will be different between TD and ASD groups.	Yes	
	(iii) TD participants will show consistency between themselves as to which items are hardest, but this will not be true for the ASD participants.	Yes^b^	

ASD: autism spectrum disorders; TD: typically developed.

a With the caveat that upper limits for response times were capped.

b For high-mentalising items only.

The second finding was that while the ASD group showed problems on this test of mentalising (in agreement with a vast literature outlining ToM-related deficits among ASDs using similar kinds of paradigms (e.g. Baron-Cohen et al., 1997; Castelli et al., 2000, 2002; Dziobek et al., 2006; Frith & Frith, 2006; Golan et al., 2007; Happé, 1994; Heavey et al., 2000)), it is questionable to assert that weak mentalising abilities *alone* can entirely explain the observed behaviour. A novel aspect of our investigation was that we administered the task in such a way that we could determine whether the ASD participants, when they failed a test item, were failing in the same way as TD participants who failed that item. We examined these patterns in multiple ways, and the results converged on a conclusion that the ASD participants did not show the same pattern of failures as the TD group. Specifically, we considered three main patterns of performance in this regard. The first was to consider if ASD and TD participants make different errors to individual test items. In other words, if TD participants tend to make an error on test item 9 by choosing the ‘irrelevant’ option, do the ASD participants also make this error on that item (when they do). We examined this by creating an index that described how ‘typical’ or ‘usual’ any particular type of error was for each different test item. The ASD participants’ typicality or normality score was significantly lower than in the TDs. In other words, when the ASD participants failed an item, they departed from making the same errors as the TDs did to that item. One of these types of error was the ‘no responses’ category. Prima facie this might be related to slow response times in the ASD group. However, the group difference in the ‘no response’ category remains significant after co-varying for general RTs, and we did not find the ASD participants as a group to be generally slower than the TDs across all items. So clearly, this warrants further investigation.

But a second and more curious finding was related to Hypothesis 2, which was that the relative proportion of different error types would be different between the TD and ASD groups. When considering the pattern of ‘incorrect’ versus ‘irrelevant’ choices made by the group, we found that the TD participants made almost three times the number of ‘incorrect’ choices compared to ‘irrelevant’ ones, but the ASD participants made only 25% more. This is a highly significant group difference. Put simply, the ASD participants were choosing the ‘irrelevant’ response option (see Tables 1 and 3 for examples) much more frequently while also choosing the ‘incorrect’ option proportionally less frequently than the TD group (see Table 5).

A further hypothesis about the ASD performances that came from recent error analyses of tests of other kinds of cognitive functions (Thiébaut et al., 2016; Wu et al., 2018) was that TD participants will show consistency between themselves as to which items are hardest, but this will not be true for the ASD participants (Hypothesis 5(iii)). This did in turn out to be that case, but only for high-mentalising test items. In other words, the TD participants showed strong agreement with each other about which test items were hardest, irrespective of whether these items were high- or low-mentalising ones. However, for the ASD group, there was markedly less agreement among participants for high-mentalising items than there was in the TD group. In this way, not only were the ASD group responses more heterogeneous compared to the TD group’s responses but they were also more heterogeneous when considering responses within groups (i.e. the ASD participants were more dissimilar to each other than were the TDs, in terms of their response choices).

The overall pattern of results here seems more complex than what would be predicted if the atypicalities in the ASD group were *only* caused by a poor ability to mentalise. One possible characterisation of the data is that when the ASD participants misunderstood what was happening in the video clips, they were not misunderstanding them in the same way as the TDs did but were instead coming to different conclusions about what they had just witnessed. However, where participants have not made a response at all, the precise reason for that absence of response will await further investigation.

Another, perhaps related, possible account for these kinds of difference in pattern of behaviour relates to the fact that the format of mentalising tasks commonly used for research (including the one used here) means that they do not only tap mentalising but also tap decision-making processes. It has been known for many years that multiple-choice formats (indeed, perhaps any kind of test format) create decision-making demands quite independently of the level of knowledge of the person. Indeed, psychometric theory is quite well developed on this (see e.g. Haladyna, 1999; F. M. Lord, 1952). For instance, to perform a typical mentalising task, the participant is first required to be able to process and understand the stimulus material. But then, they are required to give a response or judgement about that material that will involve additional high-level decision-making processes, such as criterion-setting (where the participant determines how confident in their judgement, they need to be to make a particular choice, e.g. Thiébaut et al., 2016). In some circumstances, those high-level ‘top-down’ processes may, in theory, affect how a person approaches and attends to the stimulus material, and therefore how it is understood, or at least, the conclusions that are drawn from it by the participant. There is ample empirical evidence to suggest that differences in such high-level processes can affect ASD performances on a range of social and non-social tasks (e.g. O’Hearn et al., 2008; Thiébaut et al., 2016; White et al., 2009; White, 2013), even when they are matched with the control or comparison group for IQ test performance. So one account of the heterogeneity of responding seen here in the ASD group might be that while they may be reasonably homogeneous for mentalising ability (i.e. since in part they acquired a diagnosis because of it, so were selected for a certain level), they may be more heterogeneous in their higher-level decision-making abilities (since variance in such abilities may be less closely selected for in the diagnostic procedures). It would be premature to take a firm view at this stage. But it seems a promising avenue for the future when looking at mentalising test performances to consider that a simple summary outcome measure (e.g. number of correct vs wrong items, or RTs) might contain variance attributable to more than one source and to design the tasks in such a way that the potential contribution of higher-level decision-making processes to performance could be assessed. This study also highlights one of the methodological issues with studying cognition and behaviour in ASD samples; when studying a population where within group atypicality in behaviour or responding is a key feature, the experimenter likely requires a considerable sample size to fully determine the results. Whereas where a substantial proportion of a sample fail or behave in a particular way (as with our TD group), it is much easier.

However, on the main point raised by Prof. Skuse that provided the original motivation for this study, the results of this study seem clear. It does indeed seem that people with high-functioning forms of ASD may have difficulty with following conversations between multiple people (at least, when they are presented in video clip format), even when they score well on IQ tests, as Prof. Skuse observed. Our data suggest that this difficulty is probably not due to the number of people involved in the interaction per se, but the increased mentalising demands of interactions between multiple people – it appears to be multiple minds that present the problem, not integrating multiple sources of information.
